# Young and restless: validation of the Mind-Wandering Questionnaire (MWQ) reveals disruptive impact of mind-wandering for youth

**DOI:** 10.3389/fpsyg.2013.00560

**Published:** 2013-08-27

**Authors:** Michael D. Mrazek, Dawa T. Phillips, Michael S. Franklin, James M. Broadway, Jonathan W. Schooler

**Affiliations:** Department of Psychological and Brain Sciences, University of California Santa BarbaraSanta Barbara, CA, USA

**Keywords:** mind-wandering, attention, reading comprehension, K-12 education, well-being

## Abstract

Mind-wandering is the focus of extensive investigation, yet until recently there has been no validated scale to directly measure trait levels of task-unrelated thought. Scales commonly used to assess mind-wandering lack face validity, measuring related constructs such as daydreaming or behavioral errors. Here we report four studies validating a Mind-Wandering Questionnaire (MWQ) across college, high school, and middle school samples. The 5-item scale showed high internal consistency, as well as convergent validity with existing measures of mind-wandering and related constructs. Trait levels of mind-wandering, as measured by the MWQ, were correlated with task-unrelated thought measured by thought sampling during a test of reading comprehension. In both middle school and high school samples, mind-wandering during testing was associated with worse reading comprehension. By contrast, elevated trait levels of mind-wandering predicted worse mood, less life-satisfaction, greater stress, and lower self-esteem. By extending the use of thought sampling to measure mind-wandering among adolescents, our findings also validate the use of this methodology with younger populations. Both the MWQ and thought sampling indicate that mind-wandering is a pervasive—and problematic—influence on the performance and well-being of adolescents.

## Introduction

An extensive empirical literature has demonstrated that adults mind-wander as much as 30–50% of their waking lives, often at considerable expense to ongoing performance and quality of life (Kane et al., 2007; Killingsworth and Gilbert, 2010; Schooler et al., 2011). Mind-wandering is characteristically described as the interruption of task-focus by *task-unrelated thought* (TUT; Smallwood and Schooler, 2006). Consistent with this usage, the most common and direct assessment of mind-wandering involves periodically interrupting individuals during a task and asking them to report the extent to which their attention was either on-task or on task-unrelated concerns. Yet despite the growing recognition that variation in mind-wandering represents an important individual difference measure (Kane et al., 2007; McVay and Kane, 2012; Mrazek et al., 2012a,b), research has until recently proceeded without a validated scale to directly measure trait levels of task-unrelated thought. Here we report the creation and validation of a Mind-Wandering Questionnaire (MWQ), along with novel insights made possible by the use of this scale regarding the impact of mind-wandering on the academic performance and well-being of adolescents.

Scales commonly used to assess mind-wandering lack face validity, measuring related but distinct constructs like daydreaming or behavioral errors. Perhaps the most widely used scale to assess trait levels of mind-wandering is the Daydream Frequency Scale (DDFS), also known as the daydream subscale of the Imaginal Processes Inventory (Giambra, 1995). This scale was central to one of the initial investigations linking mind-wandering to the default network (a collection of brain regions that show greater activation at rest; Mason et al., 2007). Mason and colleagues argued that recruitment of the default network during practiced tasks reflected mind-wandering in part because this activation was correlated with individuals' propensity to mind-wander as measured by the DDFS. More recently, the DDFS has been used as a measure of mind-wandering in investigations linking task-unrelated thought with the opposing construct of mindfulness (Mrazek et al., 2012b; Stawarczyk et al., 2012) and with creativity (Baird et al., 2012).

Despite the widespread tendency to use the DDFS as a measure of mind-wandering, a review of the scale's items indicates that rather than focusing on task unrelated thought, it predominantly focuses on stimulus-independent thought (e.g., When I have time on my hands I daydream … ; On a long bus, train, or airplane ride, I daydream … ; When I am not paying close attention to some job, book or TV, I tend to be daydreaming.). The DDFS therefore lacks face validity as a measure of mind-wandering, even though scores on the DDFS correlate with other measures of task-unrelated thought (Mrazek et al., 2012a,b). The key difference between the constructs of daydreaming and mind-wandering relates to the relevance of a primary task from which attention is diverted. Daydreaming refers to stimulus-independent thought that does not occur during a primary task, while mind-wandering involves a redirection of attention away from a task. Although setting precise criteria for what exactly constitutes a primary task is challenging (e.g., could taking a walk be classified as a task?), the DDFS is clearly not designed to measure stimulus-independent thought that occurs during a competing task. This distinction may hold considerable importance for ongoing research given that some individuals might daydream extensively but still have the ability to focus their attention on a primary task as needed.

Although less commonly treated as a direct measure of mind-wandering, another questionnaire used to measure lapses of attention is the Attention Related Cognitive Errors Scale (ARCES; Cheyne et al., 2006). This scale assesses the frequency of everyday mistakes that are likely to be caused by failures of attention. The ARCES has been correlated with several performance markers of inattention in the Sustained Attention to Response Task (SART), and has been interpreted as a measure of the consequences of mind-wandering (Cheyne et al., 2006, 2009). Although the ARCES provides a useful measurement of attention-related failures, it cannot provide a direct assessment of trait levels of mind-wandering because not all task-unrelated thought necessarily impairs performance and not all mistakes result from lapses of attention.

A third questionnaire that has been used to assess mind-wandering is the Mindful Attention and Awareness Scale (MAAS; Brown and Ryan, 2003). This scale measures the presence or absence of attention to and awareness of what is occurring in the present. The MAAS is used as a measure of mind-wandering based on the (often implicit) logic that mindfulness and mind-wandering are opposing constructs. Indeed, research indicates that those who report high levels of mindfulness on the MAAS tend to mind-wander less during laboratory tasks (Mrazek et al., 2012b) and that mindfulness training can reduce mind-wandering (Mrazek et al., 2013).

Although the MAAS has some ability to capture variability in dispositional mind-wandering, it also has limited face validity for at least two reasons. First, the MAAS measures inattention in contexts without a clearly delineated primary task (e.g., “I find myself preoccupied with the future or the past.”) This sort of preoccupation is not mind-wandering if it occurs in the absence of a primary task. Second, the MAAS emphasizes not only attention, but also awareness. The majority of scale items target one's tendency for reflective self-awareness or meta-awareness (e.g., “I do jobs or tasks automatically, without being aware of what I'm doing”). It is possible to complete a task without meta-awareness yet with unwavering attention.

Another related challenge associated with using the MAAS as a measure of mind-wandering is that the word *mindfulness* is ultimately varied in its meaning. Some meditative traditions have defined mindfulness as sustained non-distraction (Wallace and Shapiro, 2006; Dreyfus, 2011), whereas multifactor conceptualizations of mindfulness emphasize additional qualities as well, such as an orientation toward one's experiences characterized by curiosity, openness, and acceptance (Bishop et al., 2004; Baer et al., 2006). In fact, some specifically argue that mindfulness cannot be defined as simply the absence of mind-wandering (Grossman and Van Dam, 2011). Given this disagreement, the MAAS has limitations in its use as a trait measure of mind-wandering.

In the absence of a scale specifically designed to measure mind-wandering, researchers have relied on alternative measures of related constructs like daydreaming, attention-related errors, and mindfulness. While we developed and validated a new scale to provide researchers with a direct tool for rapidly assessing trait levels of the frequency of mind-wandering, Carriere et al. (2013) developed and conducted an initial validation of two additional mind-wandering scales: the Mind Wandering-Deliberate (MW-D) scale and the Mind Wandering-Spontaneous (MW-S) scale. These scales revealed that fidgeting is associated with spontaneous but not deliberate mind-wandering (Carriere et al., 2013). Used separately, neither the MW-D nor the MW-S reflects the overall frequency of mind-wandering. Whether their combination can be used for this purpose is still undetermined.

The MWQ presented here is intended to measure the *frequency* of mind-wandering, irrespective of whether mind-wandering is deliberate or spontaneous. Items for the MWQ were written in simple language, with the intention that this scale could also be used with adolescents. Despite the wide recognition that mind-wandering is a pervasive influence among adults, the role of mind-wandering among adolescents has received little attention. This stands in contrast to strong evidence that attention problems more generally among youth are both widespread and significant. A recent meta-analysis reviewing research linking attention problems and academic achievement found that attention problems—defined according to clinical and sub-clinical levels of symptoms associated with Attention Deficit Hyperactivity Disorder (ADHD)—were associated with a variety of academic challenges (Polderman et al., 2010). Attention problems predicted grade repetition, the need for special education, and lower scores on achievement tests.

Given that attention problems are a major challenge for young students, existing and new methodologies used to study mind-wandering might facilitate a more complete understanding of inattention among youth. Here we examine the usefulness of both the MWQ and existing measures of mind-wandering in adolescent populations. This allows us to extend the use of these methods to younger populations while also investigating whether mind-wandering—which is known to be extensive and disruptive among adults—is an equally pervasive and problematic influence among middle school and high school students.

## Experimental overview

Study 1 describes the development of the MWQ and the determination of its reliability and conceptual homogeneity in a large sample of college undergraduates. Study 2 used a separate undergraduate sample to explore the convergent validity of the MWQ with thought sampling of mind-wandering during a test of working memory capacity. In Studies 3–4, we extended the validation of the MWQ to younger populations by measuring middle school and high school students' mind-wandering using the MWQ and also with thought sampling during a test of reading comprehension. We then examined how mind-wandering was associated with reading ability, stress, self-esteem, mood, and life satisfaction. Studies 3–4 therefore provided the opportunity to (1) extend the validation of the MWQ to younger populations, (2) establish the usefulness of thought sampling as a measure of mind-wandering with younger populations, (3) investigate whether mind-wandering during testing is associated with impaired performance among adolescents, (4) examine the link between mind-wandering and several markers of quality of life, and (5) explore the hypothesis that there is a marked difference in the implications of mind-wandering that occurs during testing vs. that which occurs during daily life.

## Study 1

### Materials and methods

Three researchers independently reviewed the DDFS, ARCES, and MAAS to select items that corresponded to the following definition of mind-wandering: the interruption of task-focus by task-unrelated thought. Each researcher also contributed three novel items, and the resulting pool of items was then reviewed independently by all researchers. Five items were unanimously regarded as acceptable assessments of mind-wandering (1: I have difficulty maintaining focus on simple or repetitive work; 2: While reading, I find I haven't been thinking about the text and must therefore read it again; 3: I do things without paying full attention; 4: I find myself listening with one ear, thinking about something else at the same time; 5: I mind-wander during lectures or presentations)^1^. Following the MAAS, response options were designated along a 6-point Likert scale (1-almost never, 2-very infrequently; 3-somewhat infrequently; 4-somewhat frequently; 5-very frequently; 6-almost always).

Six hundred and sixty three undergraduates (262 male, 401 female) from the University of California Santa Barbara (UCSB) participated in exchange for course credit (mean age = 19.48 years, *SD* = 2.29, range = 18–58). As part of a larger 1-h online survey, participants completed the 5-item MWQ. For this and all following studies, participants provided written informed consent and all procedures were approved by the Human Subjects Committee of the UCSB.

### Results and discussion

Descriptive statistics for each of the five items are included in Table 1. A reliability analysis revealed a Cronbach's alpha of 0.850. This indicates good internal consistency among the items, particularly for a 5-item scale (reliability estimates tend to become larger as the number of items increases).

**Table 1:** **Descriptive statistics for each Mind-Wandering Scale item**.

**Item**	**Min.**	**Max.**	**Mean**	***SD***	**Loading**
1. I have difficulty maintaining focus on simple or repetitive work	1	6	3.43	1.23	0.714
2. While reading, I find I haven't been thinking about the text and must therefore read it again	1	6	3.95	1.13	0.737
3. I do things without paying full attention	1	6	3.63	1.09	0.845
4. I find myself listening with one ear, thinking about something else at the same time	1	6	3.80	1.10	0.842
5. I mind-wander during lectures of presentations	1	6	4.05	1.10	0.815

N = 663. Response options were designated along a 6-point Likert scale (1-almost never, 2-very infrequently; 3-somewhat infrequently; 4-somewhat frequently; 5-very frequently; 6-almost always). Loading refers to the loading of each item onto the one significant component with eigenvalue > 1 derived from the principal component factor analysis of responses to the five items.

To further test the internal consistency, we next examined the inter-item correlations. Consistently moderate inter-item correlations are preferred, as they indicate good internal reliability without highly redundant items (Clark and Watson, 1995). According to these criteria, the MWQ had good inter-item correlations (Mean: 0.540, Min: 0.439, Max: 0.684).

Given that the scale showed high internal reliability, we next examined the scale's homogeneity—the degree to which the items assess a single underlying construct—using factor analysis. Principal component factor analysis of responses to the five items revealed one significant component with eigenvalue > 1 (eigenvalue: 3.58), accounting for 63.16% of total variance. Each of the five scale items had a high loading on this component (0.714, 0.737, 0.845, 0.842, 0.815). No other significant components were extracted. These results indicate that the MWQ showed good homogeneity and that the set of items assessed a single underlying construct.

## Study 2

Having established the internal consistency and homogeneity of the MWQ in Study 1, we next examined the convergent validity of the scale among a sample of college undergraduates, specifically in relation to *thought sampling* of mind-wandering during a task. This widely used measure of mind-wandering involves periodically interrupting individuals during a task and asking them to report the extent to which their attention was on the task or task-unrelated concerns. There is a broad literature validating self-reported mind-wandering obtained through thought-sampling by using behavioral (Smallwood et al., 2004), event-related potential (ERP; Smallwood et al., 2008), and fMRI methodologies (Christoff et al., 2009). Such studies suggest that individuals are able to accurately report whether they have been mind-wandering as revealed by distinct patterns of task performance and neural activation in association with self-reported mind-wandering. Having previously established the relationship between mind-wandering measured via thought sampling and performance on the operation span task (Mrazek et al., 2012a,b) we selected this measure of working memory capacity for the initial validation of the MWQ.

### Materials and methods

Seventy-seven undergraduate students (26 male, 51 female) from the UCSB participated in exchange for course credit (mean age = 18.84, *SD* = 0.93, range = 18–22). Participants completed an automated version of the operation span task (OSPAN; Unsworth et al., 2005) with embedded thought sampling, followed by the MWQ. The OSPAN is a complex span task that presents to-be-remembered letters in alternation with an unrelated processing task (verifying the accuracy of an equation). In each trial, the to-be-remembered items were sets of 3–7 letters chosen from a pool of 12 with each individual letter presented for 250 ms. At the end of each trial, participants selected the presented items in the serial order in which they appeared. However, to allow for thought sampling during the OSPAN, five trials ended with a thought sampling probe rather than an opportunity to select the items that had appeared. Thus, there were a total of 15 complete trials and 5 trials that ended with thought probes. The thought probe asked participants to indicate to what extent their attention was either on-task or on task-unrelated concerns using a 1–5 Likert scale (1:completely on-task; 2:mostly on-task; 3:both on the task and unrelated concerns; 4:mostly on unrelated concerns; 5:completely on unrelated concerns).

Following standard procedure for this task (Conway et al., 2005), one participant with an accuracy rate of less than 85% on the unrelated processing task (including errors caused by failing to respond within a response deadline based on latencies (M + 2.5 SDs) for 15 practice items) was excluded from the analysis. Span scores were calculated as the total number of items recalled in correct serial order across all trials (Conway et al., 2005).

At unpredictable intervals during the OSPAN, five trial response screens were replaced with thought sampling probes which asked participants to indicate to what extent their attention was either on-task or on task-unrelated concerns using a 1–5 Likert scale (1:completely on-task; 2:mostly on-task; 3:both on the task and unrelated concerns; 4:mostly on unrelated concerns; 5:completely on unrelated concerns). After answering the thought probe, participants were instructed that they would begin a new trial. A mind-wandering score was computed by calculating the mean of the five thought probe responses.

### Results and discussion

Although trait levels of mind-wandering are conceptually distinct from levels of mind-wandering during challenging laboratory tasks, a positive correlation between these measures would establish the convergent validity of the MWQ with the most commonly used methodology for assessing task-unrelated thought. Indeed, MWQ scores predicted probe-caught mind-wandering during the OSPAN (*r* = 0.229, *p* = 0.047). MWQ scores also predicted OSPAN performance (*r* = −0.283, *p* = 0.013). This is consistent with the well-established negative correlation between mind-wandering and working memory capacity (Kane et al., 2007; McVay and Kane, 2009; Mrazek et al., 2012a,b) which was also true of probe-caught mind-wandering and OSPAN scores in this sample (*r* = −0.271, *p* = 0.018).

## Study 3

Study 3 sought to establish the convergent validity of the MWQ in adolescent populations while also addressing a number of unanswered questions about mind-wandering among youth. *Is thought sampling an effective measure of mind-wandering with younger populations?* Although this measure is widely used among adults, adolescents might not be able to monitor or report their conscious experience as effectively. *Is mind-wandering during testing associated with impaired performance among adolescents?* This relationship is well-established among adults, but has not yet been demonstrated among adolescents. *Is mind-wandering among youth associated with mood or quality of life*? Although mind-wandering predicts lower mood in adults (Smallwood et al., 2005, 2009; Killingsworth and Gilbert, 2010), its impact on adolescents' levels of mood, stress, self-esteem, and life satisfaction are unknown. *Finally, are there different implications for trait levels of mind-wandering vs. task-unrelated thought that occurs during testing?* We examined each of these questions in a large sample of high school students by integrating thought sampling into a test of reading comprehension and administering a set of questionnaires to assess mind-wandering and several facets of well-being.

### Materials and methods

One hundred and six female high school students from Dos Pueblos High School in Goleta, CA participated in exchange for gift cards. As the pre-testing for a larger training study, participants completed a 20-min reading comprehension test with thought sampling and a battery of scales described below. Test passages and questions were selected from standardized reading comprehension testing materials used in Alaska and Florida. Two versions matched for length, number of questions, and difficulty were created for each grade.

At unpredictable intervals during the reading test, participants were interrupted 8 times and asked to answer thought sampling probes identical to those in Study 2. After answering each thought probe, participants were instructed to resume their reading test. A mind-wandering score was computed by calculating the mean of the eight thought probe responses.

Following the reading test, participants completed the following scales: MWQ; MAAS; Satisfaction with Life Scale (SWLS); Perceived Stress Scale (PSS); Positive and Negative Affect Schedule (PANAS); and the Rosenberg Self-Esteem Scale (RSES). A composite mood score was computed for the PANAS by subtracting the negative affect sub-scale from the positive affect sub-scale.

### Results and discussion

Correlations among all measures are reported in Table 2. Mind-wandering during testing as assessed by thought sampling was negatively correlated with reading comprehension. This result indicates that high school students are able to monitor and report the focus of their attention with sufficient accuracy to predict their test performance. Trait levels of mind-wandering as measured by the MWQ were correlated with task-unrelated thought during the test of reading comprehension. Whereas mind-wandering during testing was significantly associated with worse reading comprehension, high scores on the MWQ significantly predicted worse mood, greater stress, and lower self-esteem.

**Table 2:** **Correlations among MWQ, performance measures, and scales for high school students**.

**Variable**	**MWQ**	**TUT**	**Reading**	**MAAS**	**PSS**	**RSES**	**PANAS**	**SWLS**
1. MWQ	−							
2. Reading TUT	0.233^**^	−						
3. Reading accuracy	0.153	−0.188^*^	−					
4. MAAS	−0.578^***^	−0.157^*^	−0.101	−				
5. PSS	0.278^***^	0.206^*^	−0.012	−0.402^***^	−			
6. RSES	−0.294^***^	−0.163^*^	0.065	0.285^***^	−0.437^***^	−		
7. PANAS	−0.291^***^	−0.147	−0.044	0.356^***^	−0.587^***^	0.485^***^	−	
8. SWLS	−0.124	−0.198^*^	0.162^*^	0.259^**^	−0.562^***^	0.573^***^	0.484^***^	−
Mean	3.68	1.84	0.40	3.92	2.16	2.76	0.63	4.35
*SD*	1.01	0.53	0.19	0.81	0.62	0.50	1.05	1.30

N = 156. TUT, task-unrelated thought; MAAS, Mindful Attention and Awareness Scale; PSS, Perceived Stress Scale; RSES, Rosenberg Self-Esteem Scale; PANAS, Positive and Negative Affect Schedule; SWLS, Satisfaction with Life Scale.

* p < 0.05,

** p < 0.01,

*** p < 0.001.

Having administered both the MWQ and the MAAS, we next examined which of these scales was a better predictor of mind-wandering during testing. We conducted a simultaneous regression analysis predicting mind-wandering during testing from MWQ and MAAS scores. We examined the collinearity statistics of the predictor variables and found that the tolerance of both the MWQ and MAAS were 0.67 and the VIF were 1.50, indicating that there was no evidence of a multicollinearity problem. The overall regression model was significant, *F*_(1, 153)_ = 4.467, *p* = 0.013. Only the MWQ was a significant predictor of mind-wandering during testing. An inspection of the standardized partial regression coefficients (β) and semi-partial correlations (*sr*^2^) revealed that the MWQ explained a significant amount of unique variance in mind-wandering (β = 0.214, *p* = 0.028, *sr*^2^ = 0.177), whereas the MAAS did not (β = 0.033, *p* = 0.729, *sr*^2^ = 0.028).

## Study 4

Given that the MWQ measured trait levels of mind-wandering among high school students in a way that led to a predicted and informative pattern of relationships with other measures, we sought to extend the use of this scale to a middle school sample.

### Materials and methods

Seventy-eight middle school students (35 male, 43 female) from Santa Barbara Middle School and Crane Country Day School in Santa Barbara, CA participated in exchange for gift cards (68 students in Grade 7, 10 students in Grade 6). As the pre-testing for a larger training study, participants completed the same experimental procedure as in Study 3. Test passages and questions were selected from standardized state reading comprehension testing materials used in California. Two versions matched for length, number of questions, and difficulty were created for each grade. Participants completed the five questionnaires in a counterbalanced order.

### Results and discussion

The pattern of correlations among measures in this sample of middle school students (Table 3) closely resembled results from Study 3. Mind-wandering during testing was associated with impaired reading comprehension. High scores on the MWQ predicted mind-wandering during testing, but not reading comprehension. Finally, high levels of mind-wandering on the MWQ predicted worse mood, greater stress, and lower self-esteem. These results suggest that even middle school students of 11–13 years of age can report the focus of their attention with reasonable accuracy. The similarity in findings across high school and middle school samples indicates that mind-wandering may hold similar implications across these age groups.

**Table 3:** **Correlations among MWS, performance measures, and scales for middle school students**.

**Variable**	**MWS**	**TUT**	**Reading**	**MAAS**	**PSS**	**RSES**	**PANAS**	**SWLS**
1. MWS	−							
2. Reading TUT	0.299^**^	−						
3. Reading accuracy	0.046	−0.246^*^	−					
4. MAAS	−0.541^***^	−0.225^*^	0.071	−				
5. PSS	0.303^**^	0.044	−0.029	−0.341^**^	−			
6. RSES	−0.295^**^	−0.187	−0.036	0.343^**^	−0.446^***^	−		
7. PANAS	−0.495^***^	−0.179	−0.060	0.295^**^	−0.630^***^	0.482^***^	−	
8. SWLS	−0.313^**^	−0.046	−0.110	0.367^**^	−0.560^***^	0.489^***^	0.505^***^	−
Mean	3.44	1.92	0.86	4.02	2.47	2.55	1.35	5.22
*SD*	0.96	0.66	0.13	0.80	0.82	0.17	1.08	1.42

N = 78. TUT, task-unrelated thought; MAAS, Mindful Attention and Awareness Scale; PSS, Perceived Stress Scale; RSES, Rosenberg Self-Esteem Scale; PANAS, Positive and Negative Affect Schedule; SWLS, Satisfaction with Life Scale.

* p < 0.05,

** p < 0.01,

*** p < 0.001.

## General discussion

The MWQ is a face-valid tool for rapidly assessing trait levels of mind-wandering. The scale showed high internal consistency and good homogeneity. It predicted mind-wandering assessed via thought sampling in college, high school, and middle school populations. The current research therefore suggests that the MWQ is a suitable tool for researchers to use when they are specifically interested in mind-wandering, as opposed to related constructs assessed by the DDFS, ARCES, or MAAS.

Further validation of the MWQ would be useful, particularly with respect to additional populations of interest and greater specification of the task contexts in which performance can be predicted by trait levels of mind-wandering. The present research focused on the use of this scale with undergraduate, high school, and middle school students. Further research across cultures or with more heterogeneous adult samples or special populations like youth or adults with ADHD could determine additional domains in which the MWQ could be used. Another remaining question is whether trait levels of mind-wandering assessed via the MWQ will be useful in predicting performance on laboratory tasks. High scores on the MWQ were associated with worse working memory performance among undergraduates, but the MWQ did not predict worse reading comprehension among high school or middle school students. In fact, there was a marginally significant association between more mind-wandering on the MWQ and better reading comprehension among high school students (though not in the middle school sample). It is unclear whether this discrepancy is due to the age of participants, the type of task, or some additional variable. Regardless, the present findings suggest that the MWQ cannot be used in place of thought sampling for researchers specifically interested in mind-wandering that occurs during laboratory tests of cognitive performance.

Children develop more explicit and effective metacognition as they age (Kuhn, 2000), yet the current findings suggest that children as young as 11 years of age (in 6th grade) are able to report the focus of their attention in a way that meaningfully correlates with their reading comprehension and well-being. Given the usefulness of thought sampling in characterizing the causes and consequences of mind-wandering among adults (Schooler et al., 2011), continued investigation of inattention among youth may be facilitated by the present demonstration that thought sampling can also be an appropriate method with adolescents. Both existing research and the present findings suggest that attention problems among youth are both widespread and significant (Polderman et al., 2010). More comprehensive analysis of the prevalence and impact of mind-wandering in schools are clearly warranted. For instance, mind-wandering during lectures is associated with impaired learning among college students (Risko et al., 2012), suggesting that the use of thought sampling in other high school and middle school contexts might reveal that the link between mind-wandering and impaired reading comprehension has parallels in other educational outcomes.

High levels of mind-wandering as assessed by the MWQ were associated with worse mood, greater stress, and lower self-esteem among adolescents. These results indicate that efforts to enhance the well-being of youth might benefit from greater recognition and understanding of the impact of mind-wandering on social and emotional dimensions of students' lives. The correlational findings in the present research do not indicate that mind-wandering leads to lower quality of life, but research does suggest that mind-wandering may *cause* worse mood in adults (Smallwood et al., 2009; Killingsworth and Gilbert, 2010). This suggests that additional research examining the social and emotional impact of mind-wandering among adolescents would be worthwhile. Even if mind-wandering is not directly causing stress, low mood, and poor self-esteem, it is still possible that interventions targeting any one of these outcomes might affect the others. Given that levels of mind-wandering can be influenced by interventions among adults (Mrazek et al., 2013), similar interventions for reducing mind-wandering early in life might help improve both academic performance and quality of life. Nevertheless, future research should also keep the potential benefits of mind-wandering in view. It may be that individuals are best off not eliminating mind-wandering altogether, but instead mind-wandering at the right times (e.g., when primary task demands are relatively modest) and on the right topics (e.g., on productive issues that can foster future planning or creativity).

## Author's note

Michael D. Mrazek, Michael S. Franklin, James M. Broadway, Dawa T. Phillips, and Jonathan W. Schooler are supported through United States Department of Education grant R305A110277 awarded to Jonathan W. Schooler. The content of this article does not necessarily reflect the position or policy of the US Government, and no official endorsement should be inferred.

### Conflict of interest statement

The authors declare that the research was conducted in the absence of any commercial or financial relationships that could be construed as a potential conflict of interest.

## Footnotes

^1^ Items 3 and 4 were adapted from MAAS items “I find myself doing things without paying attention” and “I find myself listening to someone with one ear, doing something else at the same time.”
